# Niche shifts and the potential distribution of *Phenacoccus solenopsis* (Hemiptera: Pseudococcidae) under climate change

**DOI:** 10.1371/journal.pone.0180913

**Published:** 2017-07-10

**Authors:** Jiufeng Wei, Hufang Zhang, Wanqing Zhao, Qing Zhao

**Affiliations:** 1 Department of Entomology, Shanxi Agricultural University, Taigu, Shanxi, P. R. China; 2 Xinzhou Teachers University, Xinzhou, Shanxi, P. R. China; Chinese Academy of Agricultural Sciences Institute of Plant Protection, CHINA

## Abstract

The cotton mealybug, *Phenacoccus solenopsis* Tinsley (Hemiptera: Pseudococcidae), is a serious invasive species that significantly damages plants of approximately 60 families around the world. It is originally from North America and has also been introduced to other continents. Our goals were to create a current and future potential global distribution map for this pest under climate change with MaxEnt software. We tested the hypothesis of niche conservatism for *P*. *solenopsis* by comparing its native niche in North America to its invasive niches on other continents using Principal components analyses (PCA) in R. The potentially suitable habitat for *P*. *solenopsis* in its native and non-native ranges is presented in the present paper. The results suggested that the mean temperature of the wettest quarter and the mean temperature of the driest quarter are the most important environmental variables determining the potential distribution of *P*. *solenopsis*. We found strong evidence for niche shifts in the realized climatic niche of this pest in South America and Australia due to niche unfilling; however, a niche shift in the realized climatic niche of this pest in Eurasian owing to niche expansion.

## Introduction

With rapidly increasing global trade and human movement, the rate of introductions has remarkable accelerated around the world [1]. Climate change is another important factor that affects the expansion of invasive species [2–3]. Considerable evidence suggests that climate change will aggravate the impacts of invasive species naturalization and subsequent invasion across communities and ecosystems in their new ranges, thereby resulting in threats to the biodiversity of native species [4–5] and the degradation of ecosystem [6], casuging significant economic losses [7–8].

The cotton mealybug, *Phenacoccus solenopsis* Tinsley (Hemiptera: Pseudococcidae), is a serious invasive species that significantly damages plants of approximately 60 families [9–10] and is widely distributed in more than 24 countries, causing substantial crop losses [11–12]. This pest is native to North America [13] and has expanded its range to areas outside the North American continent such as India, Pakistan and China in Asia [14–16], Brazil and Argentina in South American [17–18], and Australia [19]. Estimated yield losses of cotton due to *P*. *solenopsis* damage in India were 30–40% [20] and 1.4 million tons of cotton were infested in China [11]. These data suggests that *P*.*solenopsis* is a serious threat to cotton production worldwide.

Testing climatic niche conservatism and modelling habitat suitability under the scenario of climate change are necessary for developing strategies to limit the introduction as well as expansion of invasive species and to provide important ecological and evolutionary insights into species invasions [21]. Moreover, understanding the changes in species’ niches is not only a central topics in ecology and evolution but also is especially important in the study of biological invasions [22]. Many studies have suggested that climatic niche shifts are rare between the native and invasive range for numerous species [23–25]. However, some reports have shown that climatic niche shifts in invasive species are relatively frequent between European and North America [26]. Alien species survive under new climate conditions that are different from those in their native range owing to a lack of natural enemies or local adaptation when introduced into new geographic areas. Hence, testing niche changes between native and non-native ranges is crucial for understanding range expansion and the potential invasive range.

Definition of the future distribution of invasive species is very important for early detection, control and management [27]. Many studies have investigated the current worldwide or local potential distribution of *P*. *solenopsis* [11, 28–29], but the future global distribution of this pest based on MaxEnt software is few well known. In particular, on studies have used distribution records and environmental variables to predict the changes in the potential distribution of *P*. *solenopsis* under climate change or tested climatic niche shifts for *P*. *solenopsis*. Information regarding the potential distribution of this species under climate change will be indispensable for scientists and farmers around the world to develop future monitoring and management strategies.

To address such issues, two objectives were addressed in this paper: 1) Mapping the current and future potential global distribution of *P*.*solenopsis*; 2) Testing the climatic niche conservatism between its native and invasive ranges.

## Materials and methods

### Distribution data

Species locality data for *P*. *solenopsis* Tinsley were derived from the Global Biodiversity Information Facility (GBIF) database (http://www.gbif.org, accessed November 2016), ScaleNet (http://scalenet.info/, accessed November 2017) and published studies (S1 Table).

Geo-coordinates for each chosen point were either referenced from information given in the literature or by using Google Earth. All occurrence records were checked for accuracy prior to use. A total of 334 georeferenced occurrences were assembled in the initial study.

**Fig 1:**
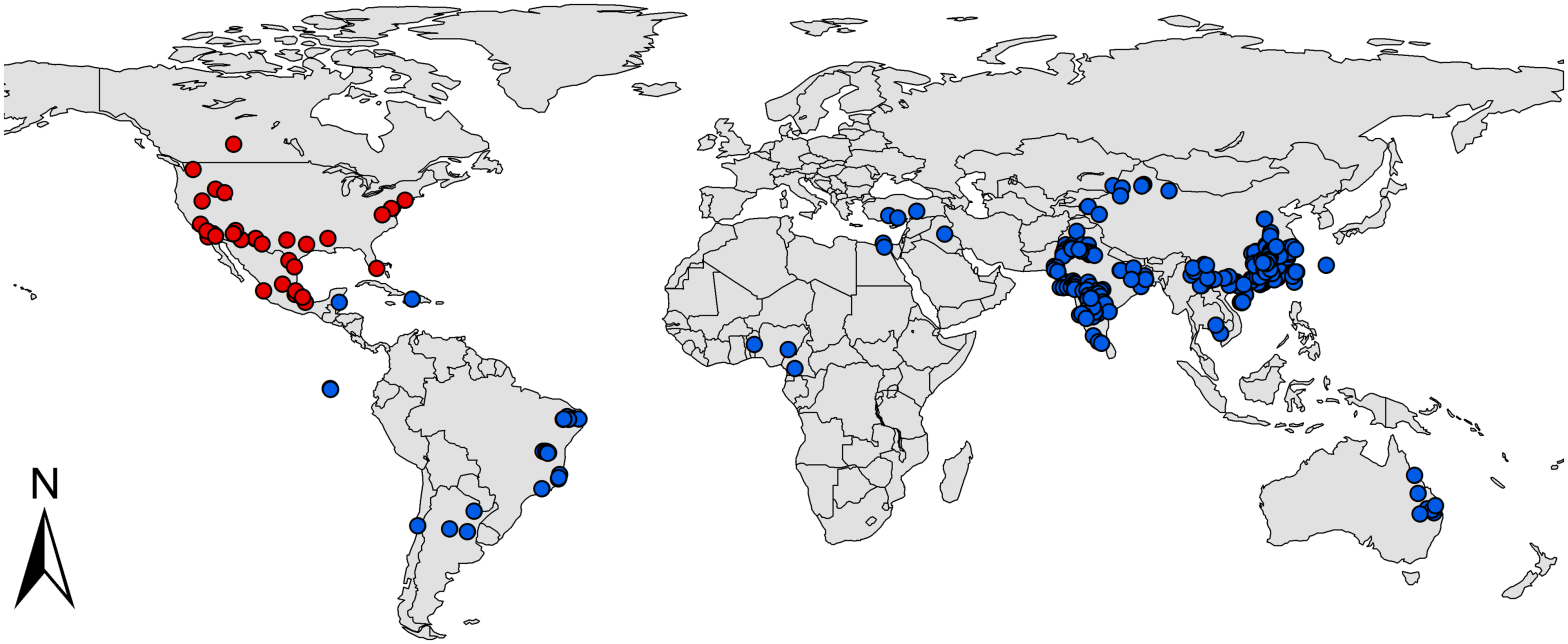
Native and invasive localities of *P*. *solenopsis* used in current modeling. Red dot represent native localities and blue dot represent invasive localities. The base map was created with Natural Earth Dataset (http://www.naturalearthdata.com/).

Occurrence records are often biased towards areas that are easily accessible or near cities or other areas of high population density [30]. Thus, to remove the spatial autocorrelation and sampling bias, we created a grid of 5×5 km cells and randomly selected a single point from each cell with one or more sampling points. After filtering, 201 locations remained, including the native North American range (29 points) and the invaded regions in Eurasia (144 points), Australia (6 points), Africa (5 points) and South America (17 points). The distribution of the locations used in the study is presented in Fig 1 and S2 Table.

### Climatic variables

In a general way, climatic factors play a much more important role in determining the potential distribution of species than topographical factors at a large scale [31]. Other non-climatic factors, such as bionomics and occupancy dynamics, might also operate but at a local scale [32]. Therefore, climatic factors are widely used as predictior variables to model potential species distribution ranges at a global scale with coarse resolution.

Current climatic variables were download from the WorldClim database, version 1.4 [30] (http://www.worldclim.org/). These climatic data represent minima, maxima and average values of monthly, quarterly, and annual ambient temperatures as well as precipitation values recorded between 1950 and 2000. All climatic variables had a spatial resolution of 2.5 arc min (approx. ~ 5 km resolution at the equator).

Multicollinearity among predictor variables may hamper the analysis of species-environment relationships because ecologically more causal variables can be excluded from models if other correlated variables explain the variation in the response variable better in statistical terms [33]. Hence, we used Pearson’s correlation coefficient for each pairwise comparison of all 19 climatic variables to identify and remove highly correlated variables (r≥|0.85|) (S3 Table). Multicollinearity was assessed using ENMTools version 1.0 [34]. Seven remaining predictor variables, best describing the availability of water and energy, were subsequently processed: mean diurnal range (mean of monthly (mean monthly maximum temperature—mean monthly minimum temperature)) (BIO2); Isothermality (BIO2/BIO7) (*100) (BIO3); Mean Temperature of the Wettest Quarter (BIO8); Mean Temperature of the Driest Quarter (BIO9); Precipitation Seasonality (Coefficient of Variation) (BIO15); Precipitation of Warmest Quarter (BIO18); Precipitation of Coldest Quarter (BIO19).

To predict the future potential distribution of *P*. *solenopsis* on a global scale, Hadley Global Environment Model 2-Atmosphere Ocean (HADGEM2-AO) representing simulations for four representative concentration pathways (RCP 2.6, RCP 4.5, RCP 6.0, RCP 8.5) for 2050 and 2070 were obtained from the fifth assessment of the Intergovernmental Panel for Climate Change [35].

The selected RCPs represent four possible greenhouse gas emission trajectories ranging from low (RCP2.6) to high (RCP8.5) corresponding to increases in global radiative forcing values in the year 2100 relative to preindustrial values (2.6, 4.5, 6.0 and 8.5 w/m^2^, respectively) [36]. Because extreme climatic variations may have a greater influence on the geographical distribution pattern, we selected climate projections for simulation in four climate change scenario/year combinations: RCP 2.6–2050 (average for the years 2041–2060 under scenario RCP 2.6), RCP 8.5–2050 (average for the years 2041–2080 under the scenario RCP 8.5), RCP 2.6–2070 (average for the years 2061–2080 under the scenario RCP 2.6), and RCP 8.5–2070 (average for the years 2061–2080 under the scenario RCP 8.5), these data all provided the best representation for future climate. Future climate projection data were downloaded from the World Climate Database (http://www.worldclim.org/). All data used for the ENM had a spatial resolution of 5 km (2.5 arc-min).

### Modelling approach

The potential distributions of *P*. *solenopsis* were modelled using the maximum entropy approach in MaxEnt (version 3.3.3k) [37] software. In recent years, many modelling approaches have been developed for use in biodiversity research [38], conservation biology [31] and invasion biology [39], such as: GLM, GAM, BRT, BIOCLIM, CLIMEX, GARP and MaxEnt [40]; however, MaxEnt has been shown to have the best-performing model using small, present-only datasets based on recent comparisons among these software [41]. MaxEnt has been used to predict the potential distributions of species under climate change, such as giant African snails (*Achatina fulica* Férussac, 1821) [42], Eastern grey squirrels (*Sciurus carolinensis* Gmelin, 1788)[43] and orchids (*Epipactis helleborine*) [44].

The selection of MaxEnt was based on the following reasons: 1) MaxEnt requires presence-only data [45]; 2) compared to other approaches, MaxEnt is relatively better than other modelling software [45]; 3) MaxEnt has been found to be robust in modelling low numbers of occurrences [46–47]. MaxEnt estimates the probability distribution of species presence by comparing the environmental conditions across known occurrences of the species from the target landscape or model background [48]. The parameters of MaxEnt are set as follows: linear, quadratic, product, threshold and hinge methods were used to generate the feature types. The logistic output was used in MaxEnt, which generates a continuous map with an estimated probability of presence between 0 and 1. A jackknife test was used to estimate the significance of the contribution of each variable to the model. The convergence threshold (10^−5^), maximum iterations (5000) and max number of background points (10000) were used to run the model. The MaxEnt model was created based on the 10-fold cross-validation method.

### Model evaluation

The area under the curve (AUC) of the receiver operating characteristic (ROC) which was created by MaxEnt, was used to estimate the performance of the model [49]. AUC values range from 0 to 1, where a value of <0.5 can be interpreted as a random prediction. An AUC value between 0.5 and 0.7 indicates poor model performance, 0.7–0.9 indicates moderate performance, and >0.9 indicates high performance [50–51].

To improve the displays of prediction in this study, the continuous suitability maps predicted by MaxEnt were converted into suitable/unsuitable areaa (binary habitat) by appling a threshold value. Here, maximum training sensitivity plus specificity was used to define habitat and non-habitat for *P*. *solenopsis*. This threshold has been used in many primary studies [52–56].

### Niche overlap test

Given the above study, the niche shifts were evaluated between North America (native range) and Eurasia (invaded range); meanwhile, niche shifts between North America (native range) and Australia (invaded range) and between North America (native range) and South America (invaded range) were also estimated.

The method used was proposed by Broennimann *et al*. [57], who used principal component analysis (PCA) to transform the environmental space of the investigative environmental variables into a two-dimensional space defined by the first and second principal components [24]. Then, the two-dimensional environmental space was projected onto a 100×100 PCA grid of cells bounded by the minimum and maximum PCA values in the background data. In this step, a kernel function was used to smooth the climatic space defined in the gridded PCA climatic spaces based on the first two PCs [23]. Third, statistical tests of niche equivalency and similarity were implemented [58]. If the niche overlap value falls outside the 95% confidence interval of the null hypotheses, equivalency of the two niches can be rejected. For the similarity test, a *p* value >0.05 indicated that the niches were no more similar than expected by chance. The Schoener’s D metric was used to measure the niche overlap, which varies from 0 (no overlap) to 1 (overlap). Niche unfilling and niche expansion were also calculated to provide a more complete depiction of niche shifts. Niche expansion occurs when a species colonizes environmental conditions in its invaded range that could be occupied but are absent in its native range, while niche unfilling occurs when a species fails to colonize climates in the invaded range that are occupied in the native range.

All GIS analyses were performed using ArcGIS version 10.2.1 (ESRI). The “ecospat” package in R was used to implement this analysis (https://www.r-project.org/).

## Results

### Model performance for potential distribution

**Fig 2:**
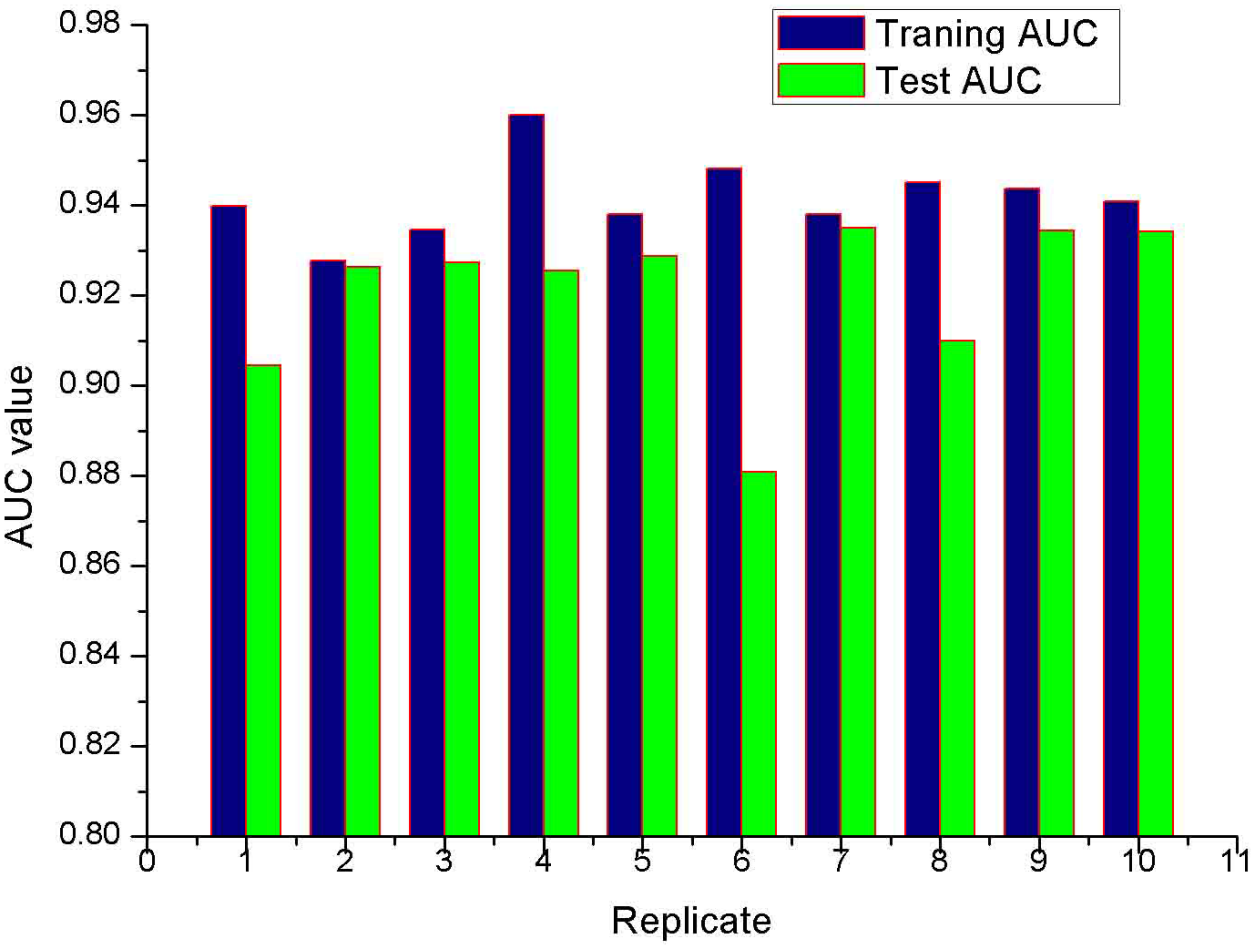
Test and training AUC value of ten-fold cross-models. Blue represent traning AUC and green represent test AUC.

The model performance for *P*. *solenopsis* was better than random, with a mean training AUC value of 0.942 (ranging from 0.927–0.948) and a test AUC value of 0.92 (ranging from 0.88–0.935) (Fig 2); therefore, the model performed well in predicting the suitable habitat area for the species. A “maximum training sensitivity plus specificity” threshold value of 0.272 was obtained from the 10^th^ percentile training presence occurrence of the species. The relative contributions of each environmental variables to the MaxEnt model are shown in Table 1. BIO8 (mean temperature of wettest quarter) (29.8%) and BIO9 (mean temperature of driest quarter) (26%) were the most important environmental variables determining the distribution of *P*. *solenopsis*. These two factors could explain 55.8% of the model. However, BIO18 (Precipitation of Warmest Quarter) (4%) and BIO19 (Precipitation of Coldest Quarter) (9.6%) could contributed 13.6% of the model. It appeared that thermal condition were more important than other variables in creating the map of this pest. The suitable habit areas for *P*. *solenopsis* were reclassified into four levels: unsuitable habitat (<0.272), low habitat suitability (0.272–0.400), moderate habitat suitability (moderate risk) (0.400–0.600), and highly habitat suitability (high invasion risk) (0.6–1.0).

**Table 1:** Relative contribution of each environmental variables to MaxEnt model.

Environment variables	relative contribution
Bio8	29.8%
Bio9	26%
Bio3	20.5%
Bio19	9.6%
Bio18	4%
Bio2	3.7%

### Current invasion pattern

**Fig 3:**
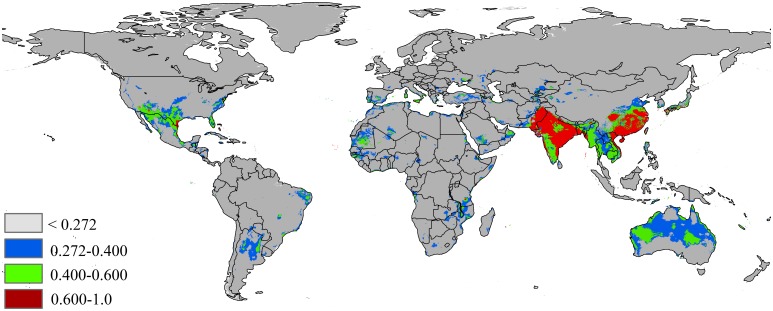
Global potential distribution map of *P*.*solenopsis* based on current climate variables. Gray = unsuitable habitat area; Blue = low habitat suitability area; Green = moderate habitat suirability area; Red = highly habitat suitability area. The base map was created with Natural Earth Dataset (http://www.naturalearthdata.com/).

The potential distribution map based on the current climate and occurrence records of *P*. *solenopsis* is shown in Fig 3. The current model shows most areas of Asia and Australia have suitable environmental conditions for the invasion species of *P*. *solenopsis*. The other continents, Africa and North America, have scattered distributions of *P*. *solenopsis* based on the current maps. In Asia, China, India, Japan, Laos, Vietnam, Bangladesh and Pakistan have environments with high suitability for this pest. The total potential area of invasion based on current environmental variables is 12,690,522 km^2^, of which 2,802,672 km^2^ (22% of the total potentially invaded area) has highly habitat suitability (high risk), and 3,780,504 km^2^ (29% of the total potentially invaded area) has moderate habitat suitability (Table 2).

**Table 2:** Area with suitability under different climate scenarios.

Range	Current	RCP2.6–2050	RCP8.5–2050	RCP2.6–2070	RCP8.5–2070
0.272–0.4	6,107,304	7,063,110 (15.6%)	7,494,353 (22.7%)	7,619,742 (24.7%)	7,876,008 (28.9%)
0.4–0.6	3,780,504	3,533,814 (-6.7%)	4,077,072 (0.7%)	4,138,974 (0.9%)	4,250,322 (12.4%)
0.6–1	2,802,672	4,116,978 (46.8%)	4,044,888 (44.32%)	4,049,946 (44.5%)	3,978,630 (41.9%)
0.272–1 (in total)	12,690,522	14,713,902 (15.9%)	15,616,313 (23%)	15,808,662 (24.5%)	16,104,960 (26.9%)

**Fig 4:**
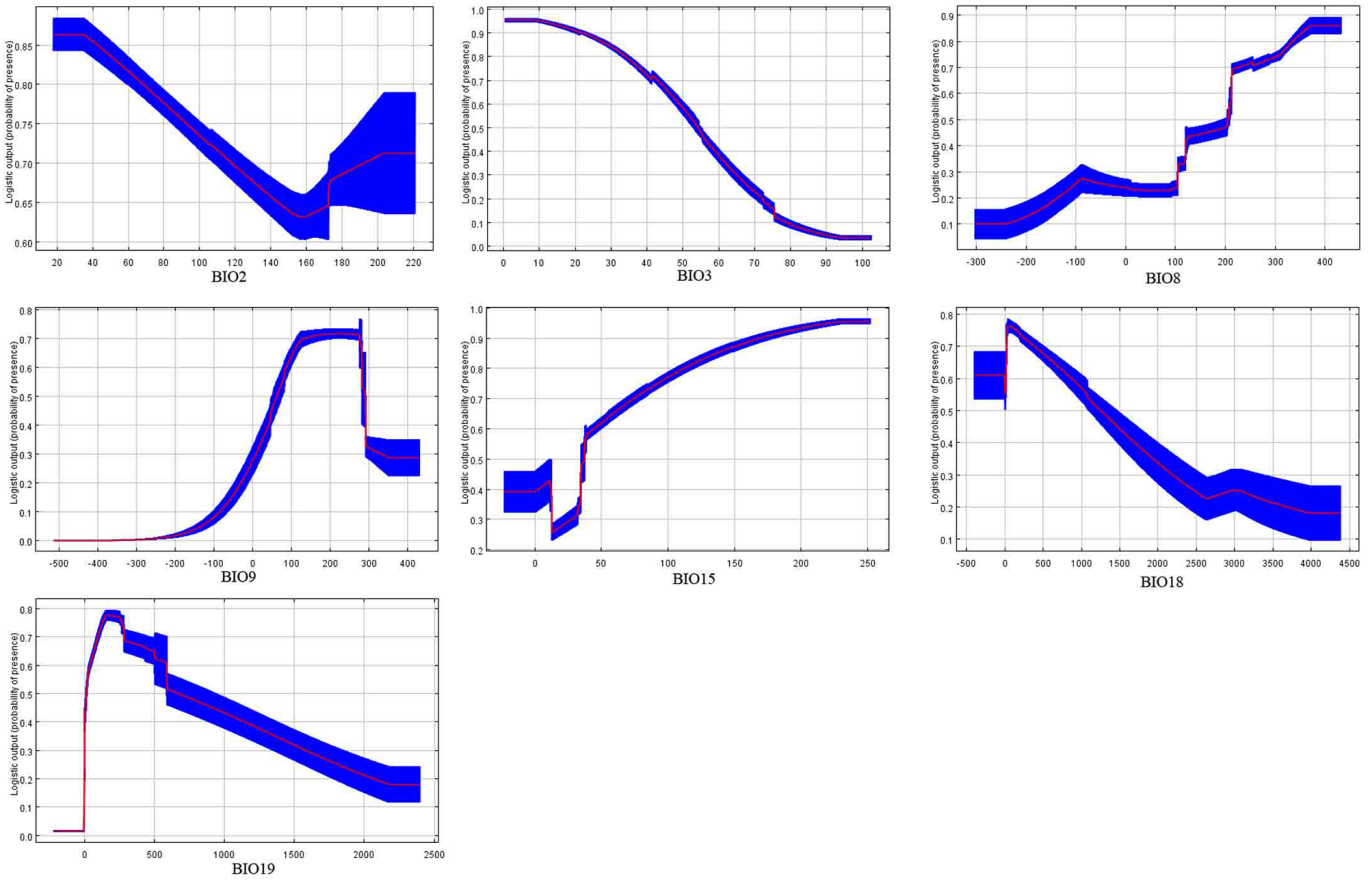
Response curves showing the relationships between the probability of presence of P. solenopsis and seven bioclimatic variables. Values shown are average over 10 replicate runs: blue margins show ±SD calculated over 10 replicates.

Based on the respondse curves (Fig 4), we found that the climate conditions associated with highly habitat suitability were 2–16°C for BIO2, 0–4.9 for BIO3, 22–37°C for BIO8, 8–19°C for BIO9, 48–230% for BIO15, 0–4000 mm for BIO18 and 30–2200 mm for BIO19.

### Future invasion risk

**Fig 5:**
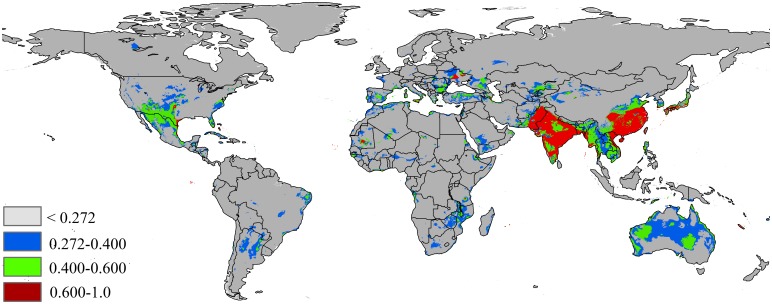
Future species distribution models under climate change scenarios RCP 2.6–2050. Gray = unsuitable habitat area; Blue = low habitat suitability area; Green = moderate habitat suirability area; Red = highly habitat suitability area. The base map was created with Natural Earth Dataset (http://www.naturalearthdata.com/).

The MaxEnt models based on RCP2.6 emission scenarios for the potential distribution of *P*. *solenopsis* in 2050 are presented in Fig 5 and Table 2. The predicted area gains in the area of suitable habitat is 14,713,902 km^2^, which is an increase of 15% over the current suitable habitat area. The highly suitable area expands to 4,116,978 km^2^ (ca. increase of 46.8% over the current highly suitable areas), including more of Asia, such as Burma, south Korea, the Philippines, China, India, Japan, Laos, Vietnam, Bangladesh, Pakistan and Ukraine in Europe. In addition, the suitable habitat was increased in North America (native area).

**Fig 6:**
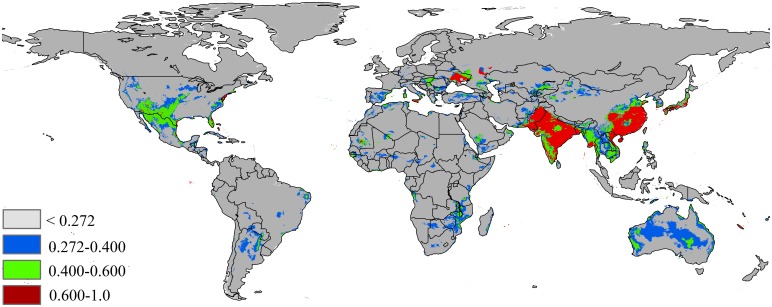
Future species distribution models under climate change scenarios RCP 2.6–2070. Gray = unsuitable habitat area; Blue = low habitat suitability area; Green = moderate habitat suirability area; Red = highly habitat suitability area. The base map was created with Natural Earth Dataset (http://www.naturalearthdata.com/).

The potential distribution of *P*. *solenopsis* under the RCP 2.6 for 2070 is presented in Fig 6 and Table 2; there was an increase of 24% over the current area of suitable habitat area, expanding to 15,808,662 km^2^. The highly suitable areas will expand to 4,049,946 km^2^ (ca. increase of 44.5% over current highly suitable areas) under this climate scenarios.

**Fig 7:**
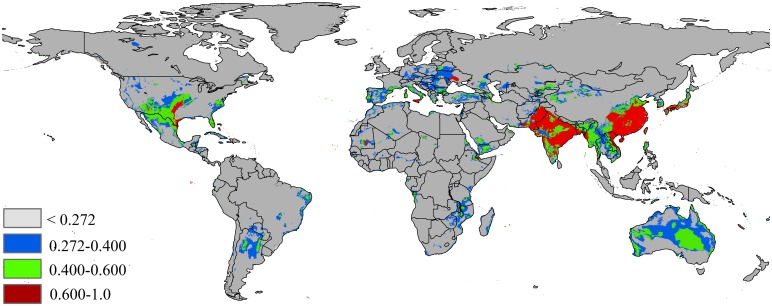
Future species distribution models under climate change scenarios RCP 8.5–2050. Gray = unsuitable habitat area; Blue = low habitat suitability area; Green = moderate habitat suirability area; Red = highly habitat suitability area. The base map was created with Natural Earth Dataset (http://www.naturalearthdata.com/).

**Fig 8:**
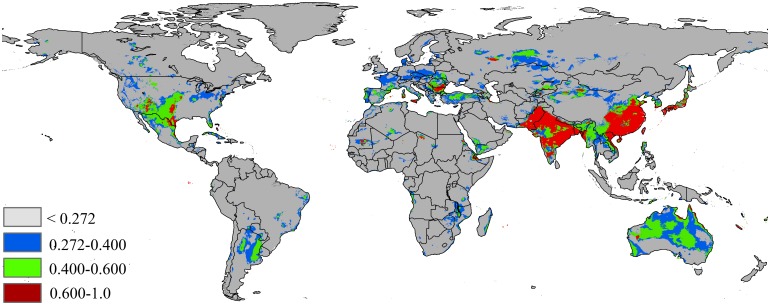
Future species distribution models under climate change scenarios RCP 8.5–2070. Gray = unsuitable habitat area; Blue = low habitat suitability area; Green = moderate habitat suirability area; Red = highly habitat suitability area. The base map was created with Natural Earth Dataset (http://www.naturalearthdata.com/).

Under RCP 8.5 for 2050, the model-predicted area of suitable habitat was 15,616,313 km^2^, which is an increase of 23% over the current suitable habitat area (Fig 7, Table 2). In this scenarios, the highly potential suitable area in Asia will expand further, to 4,044,888 km^2^ (ca. increase of 44.3% over the current highly suitable areas). Under another climate scenarios, RCP 8.5–2070 (Fig 8, Table 2), the highly suitable areas was smaller than the projection for future climate scenario RCP 8.5–2050 (ca. 2% decrease), reduced to 3,978,630 km^2^. Although the highly suitable declined, the total suitable habitat area still increased, expanding to 16,104,960 km^2^ (ca. increase of 26.9% over the current highly suitable areas).

**Fig 9:**
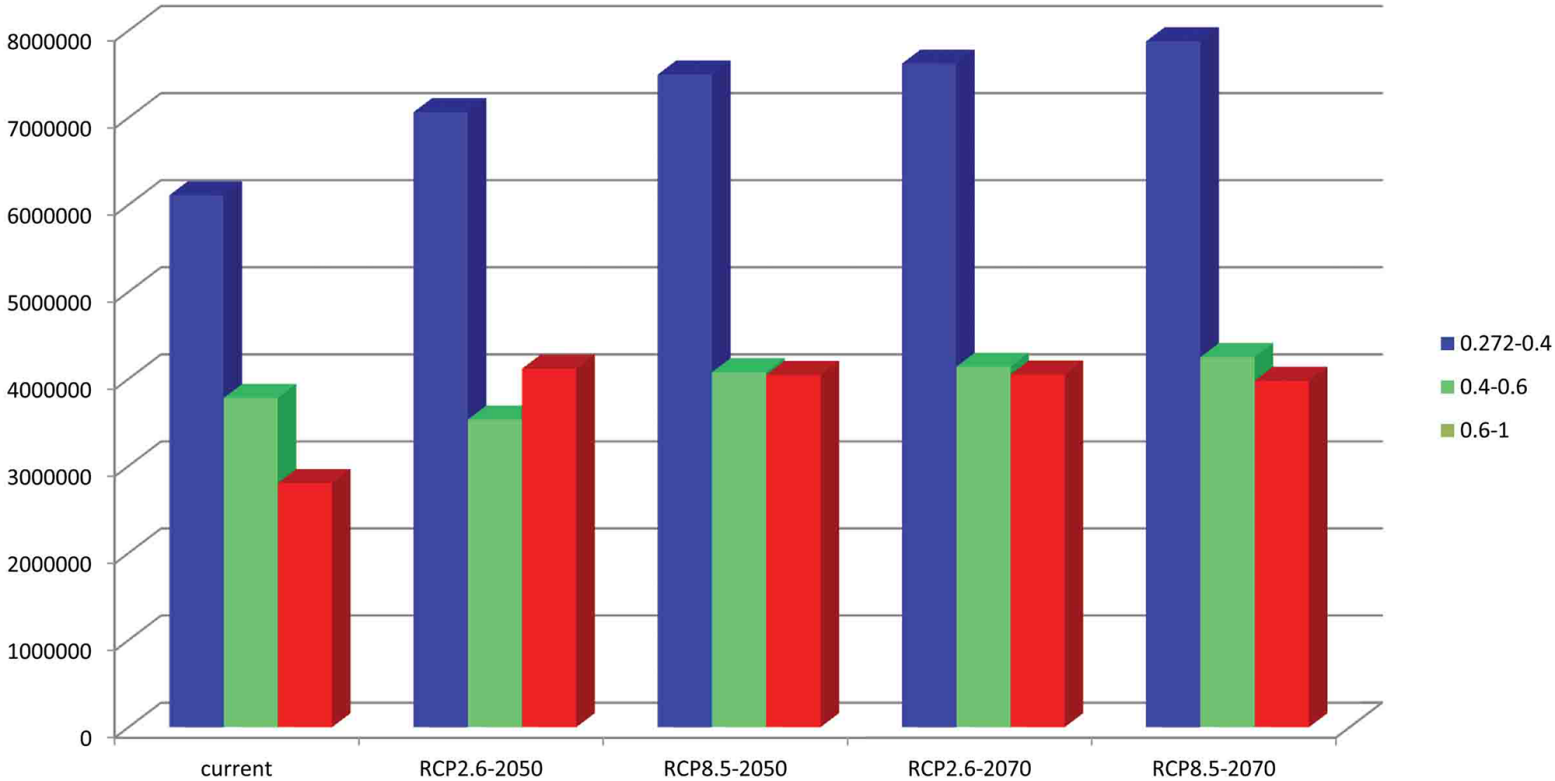
Comparison of future potential suitable habitat areas for *P*. *solenopsi* by MaxEnt for time frames on different climate scenarios. Blue = low habitat suitability area; Green = moderate habitat suirability area; Red = highly habitat suitability area.

In brief, the areas of low habitat suitability and moderate habitat suitability are expected to increase over time under climate change. The area of high habitat suitability reaches a peak under RCP 2.5–2050 and slightly decreases under other climate scenarios (Fig 9, Table 2).

### Niche overlap test

**Fig 10:**
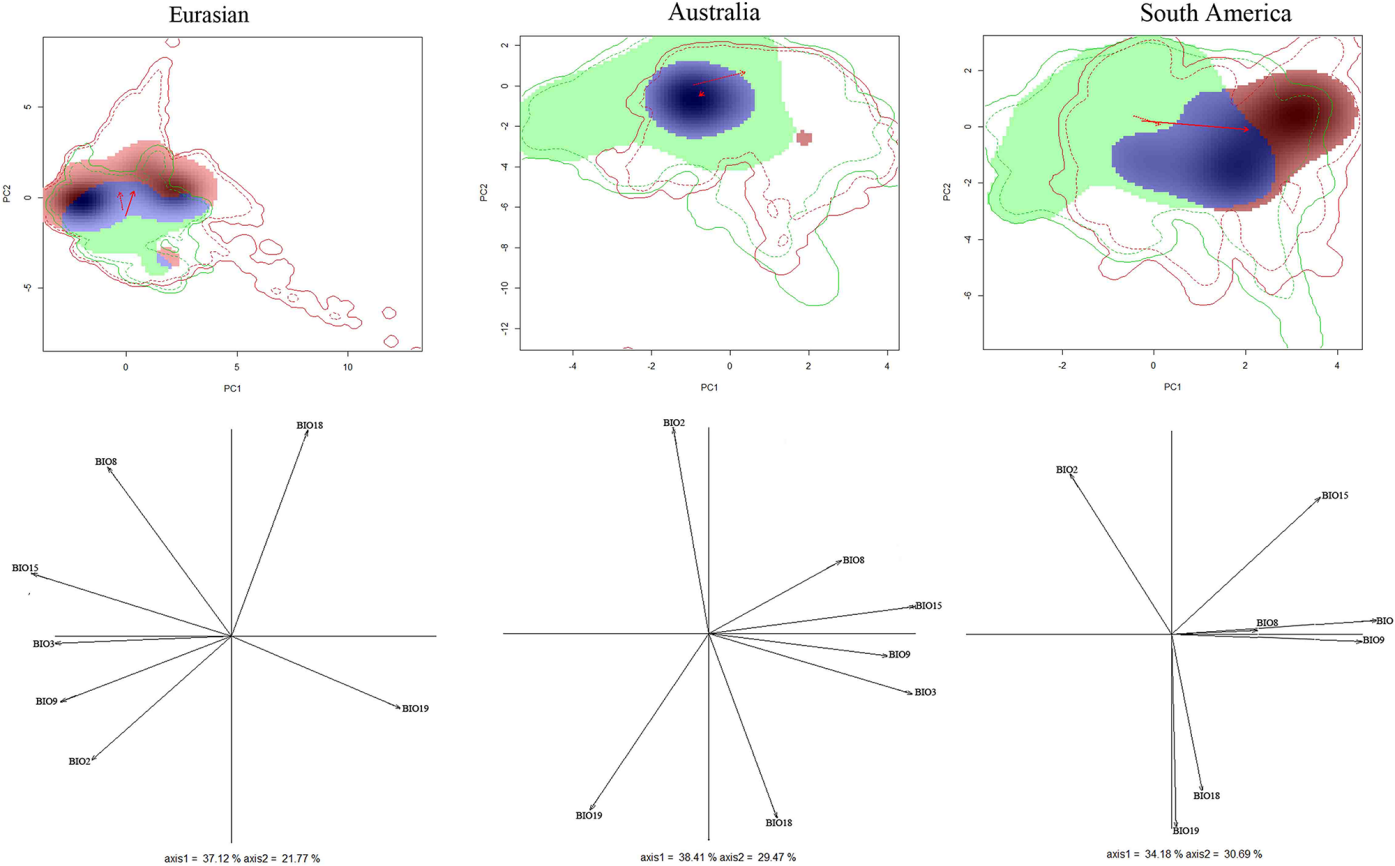
Native and invasive niches of *P*. *solenopsis* in different regions; multivariate climatic space was calculated using PCA-env method. PC1 and PC2 represent the first two axes of the principal competent analysis (PCA). The green and red shadings represent density of species occurrences in different regions; blue represents overlap. Solid and dashed lines show 100% and 50% of the available (background) environment. The red arrows show how the center of the climatic niche for *P*. *solenopsis* (solid) and background extent (dotted) has moved between two ranges.

The values from the niche overlap analysis are shown in Fig 10 and Table 3. Niche overlap between the native and non-native ranges of *P*. *solenopsi* was very low in all analyses. There was only 2% niche overlap between the native range and Eurasia (Schoener’s D = 0.02) and 9% between native range and Australia (Schoener’s D = 0.09). The results also show the native and South America as having only 8% niche overlap (Schoener’s D = 0.08). Niche unfilling was more prevalent than niche expansion except in the native vs. Eurasian comparison. In Australia, there was little evidence of the expansion of the species’ realized niche into climates that are available in the species’ native range (1.2%) and powerful evidence of niche unfilling (45.6%) (Table 3). In South America, more than 39% of the species’ invaded niche exists in climates that are not occupied in its native range, and 62.4% of the species’ native niche remains unfilled. However, in the most important and broad areas of distribution of *P*. *solenopsis*, Eurasian, there were stronger evidence of niche expansion (44.9%) and relatively weaker evidence of niche unfilling (35%). The null hypothesis of niche equivalency test was rejected for all comparisons between the native and invasive range. The results of the comparisons between the native and invasive areas indicate that this invasive species has undergone significant alterations to its environmental niche during the invasion process, as all niche equivalency was rejected. By contrast, the evaluation of niche similarity yielded no significant results from the two comparisons, which were the native range vs. South America and the native range vs. Eurasian, leading to non-rejection of the null hypotheses of niche similaritiy due to change. This result suggests that in both Eurasia and South America the occupation by this species does not follow the pattern expected by native niche requirements and seems to be random. However, the niche similarity tests showed that the niches of this pest in Australia are more similar to the niches in the native region than would be expected by chance.

**Table 3:** Result of niche overlap analysis.

Area	Schoener’s D	Similarity	Equivalency	Expansion	Unfilling	Stability
Native vs. Eurasian	0.027	ns	P < 0.05	0.449	0.350	0.550
Native vs. Australia	0.094	P = 0.03	P < 0.05	0.012	0.456	0.987
Native vs. South America	0.081	ns	P < 0.05	0.396	0.624	0.603

## Discussion

### Change in habitat suitability

The cotton mealybug is native to North America and has already invaded to other continents around the world. The map of its predicted distribution suggests that climatic changes would affect the worldwide distribution of this invasive cotton mealybug. The model shows that there would be an expansion of highly and moderately suitable habitats in both native and invasive areas. The model predicted large amounts of suitable areas in terms of climate around the world outside of the native range. The occurrence of sub-suitable areas on the current distribution map of *P*. *solenopsis* suggests that these areas were restricted by unappreciated biological conditions as a result of natural enemies, barriers to spread or local adaption. However, these areas are expected to changed over time with increasingly frequent human development and climate warming. This pest is already causing vast losses in cotton-producing areas of India [20], China [11] and Pakistan [15]. Interestingly, our map suggests that the western of Australia contains have suitable habitat for this pest; however, only five distribution sites were recorded in eastern Australia in current papers. Therefore, strict quarantine measures are needed to be enforced across Australia for reduce the spread from the east of Australia. Essentially, the present study revealed that this pest may further damage the cotton production in other areas due to the climate change, including Australia, Uzbekistan and Cambodia. The potential distribution map of this pest will be beneficial in developing monitoring strategies to detect future infestations in currently uninfested regions.

Previous studies predicting the potential distribution of *P*. *solenopsis* under climate change have focused on local areas (India) [28] and only one climatic variable (e.g., temperature) using a temperature-driven phenology model with GIS [29]. However, in the present paper, the potential distribution areas of *P*. *solenopsis* around the world were predicted based on the MaxEnt model and 7 climatic variables. The current study have resulted in the creation of a more accurate potential map under current and future climate because these environmental factors could more clearly reveal the survivabiligy of the pest. In addition, our habitat suitability map are roughly corresponds to the map created by the CLIMEX model under current climate conditions [11]. However, our study differs substantially in terms of both methodology and some habitat suitability areas. Compared to our map, the study of Wang *et al* may be overestimate or underestimate the extent of suitable habitat around the world. Several studies [59–60] suggest MaxEnt model was slightly more accurate than the CLIMEX model, which could be due to it directly used species presence locations and finer resolution climatic dataset. However, CLIMEX use relatively simple functions to model species responses to climatic factors and coarse resolution climatic dataset. In addition, the sampling spatial bias usually leads to environmental bias because of the over-representation of certain environmental features of the more accessible and extensively surveyed areas. Nevertheless, Wang *et al* did not take into account sample bias in their study. Another important factor is that only native distribution sites were employed in CLIMEX. However, the result of Shabani and Kumar [61] suggest, it is best to have entire data records rather than only native data in species distribution modeling. The map of potential distribution range of species will be distorted when only native species distribution sites are considered in species distribution moedlling.

Our model also indicates several environmental variables that explain the current distribution of *P*. *solenopsi* throughout the world. Temperature is the most important climatic variables defining the current global distribution of *P*.*solenopsis* and can explain 55.8% of this distribution model. This agrees with previous research that has shown that the population growth of this pest was mainly affected by temperature [11]. The male was unable to pupate, and the female having a comparatively very short life span, was unable to produce eggs when the temperature was above 40°C in a study of *P*. *solenopsis* in the laboratory [62]. Our model suggested an increase in the population of this pest with a mean temperature of 22–37°C in the wettest quarter and a mean temperature of 8–19°C in the driest quarter as the favoured temperature range, which is also similar to the results of Fand and his colleagues [29].

The current and future potential distribution for *P*. *solenopsis* were forecasted in current studies, but there are some factors that limit the accuracy of the prediction in our work. In current paper, the model only considered the abiotic factor which is climate variable, however, other biotic factors, such as host-plant availability. Cotton is one of the hosts of the pests, and their distribution seriously affects the distribution of pests. In addition, human-mediated transport for tourism or trade provides introduction route and therefore increase the risk of invasive species. Various reports suggested increases of biological invasions in accordance with the industrial revolution [63]. we suggest that special quarantine measure should be taken to limit potential invade for suitable region for future infestations by human activities such as cultivation and agriculture product importation. Furthermore, biotic factors, such as interspecific interactions also affect the potential range of model [64]. In addition, other important variables to include in future models chou be geographic barriers, land use, habitat loss and dispersal ability.

### Niche conservatism test

The present study is first to compare the niche changes for the invasive species *P*. *solenopsis* across multiple continents. Our study provides strong evidence of niche shifts of between native and non-native range for this pest, which implies that *P*. *solenopsis* has the potential to invade novel areas. Climatic niche shifts have been demonstrated in several invasive species, including plants [31,23, 65] and animals [24–25,66]. Our results are in agreement with these previous studies in regard to climatic niche shifts. Here, all comparisons suggested that the niche of this pest had a low overlap. This indicates that their niche was adapted to different environmental conditions in the non-native range.

The results also show that the niche shift in the realized niches of *P*. *solenopsis* in Australia and South America are mainly due to niche unfilling, suggesting that dispersal barriers or interspecific interactions might be constraining this the pest to a narrower realized niche than is physiologically possible and moreover, also suggesting that this pest is still in a colonization phase in these two areas. Indeed, interspecific interaction, such as species displacement play an important role in the invasion of alien species [67,68]. On the other hand, the lack of species displacement between alien species and native species or other alien species in colony during initial establishment may have brought advantages to the alien species. If the expansion of invasive species is limited, it may be affected by species displacement. *Liriomyza trifolii* (Burgess) is a highly invasive species that has become established in agricultural and ornamental crops throughout the world by a decade years research[67]. It had displaced *L*.*sativae* to become the predominant pest species in southern China. Thus, the niche expansion of *L*.*sativae* might be limited by the niche of *L*. *trifolii*.

By contrast, the niche shift of *P*. *solenopsis* in Eurasia exhibits very high niche expansion values (44.9% niche expansion, 35% niche unfilling). This suggests that species with narrower climatic niches in the native range show significantly higher rates of niche expansion in the alien range. Moreover, this result also shows that the distribution of this pest in Eurasia is not as strictly associated with climate as it is with host-plants, such as cotton. Cotton is one of this pest’s most favored hosts. Pakistan, India and China are the important cotton-cultivating areas around the world [11, 15, 69]. These locations provide enough resources for the survival of this invasive species. The non-climatic environment might explain niche expansions in the alien range in other studies [25, 70], but it does not facilitate the understanding of the phenomenon in the current study. The current study focuses on different areas inhabited by only one species. South America is also an important cotton-producing area. Residence times are an another interpretation of niche expansion, as species may not have enough time to fill their potential niches [56, 71]. Thus, the residence time might be shorter in Eurasia than other continents. In other words, the time of introduction in may have been earlier in South America and Australia than in Eurasian.

## Supporting information

S1 Table References used to compile the dataset. (DOC)

S2 Table The distribution of the locations used in the study. (XLS)

S3 Table Correlation analysis of environmental variables. (DOC)
