# Supplementary material for: Niche shifts and the potential distribution of Phenacoccus solenopsis (Hemiptera: Pseudococcidae) under climate change
Source: PLoS One. 2017 Jul 10;12(7):e0180913. doi: 10.1371/journal.pone.0180913 (PMC5507313; doi:10.1371/journal.pone.0180913)
Supplement: S1 Table — (DOC) [file pone.0180913.s001.doc]

References used to compile the dataset

1. Abdul-Rassoul MS, Al-Malo IM, Hermiz FB. 2015. First record and host plants of Solenospis Mealybug, *Phenacoccus solenopsis* Tinsley, 1898 (Hemiptera: Pseudococcidae) from Iraq. Journal of Biodiversity & Environmental Sciences. 7 (2): 216-222.

2. Arif MI, Rafiq M, Ghaffar A. 2009. Host plants of Cotton Mealybug (*Phenacoccus solenopsis*): A new Menace to Cotton Agroecosystem of Punjab, Pakistan. International Journal of Agriculture & Biology. 11:163-167.

3. Culik MP, Gullan PJ. 2005. A new pest of tomato and other records of mealybugs (Hemiptera: Pseudococcidae) from Espírito Santo, Brazil. Zootaxa. 964: 1-8.

4. Culik MP, Martins DS, Ventura JA, Peronti ALBG, Gullan PJ, Kondo Takumasa. 2007. Coccidae, Pseudococcidae, Ortheziidae, and Monophlebidae (Hemiptera: Coccoidea) of Espirito Santo, Brazil. Biota Neotropica. 7(3): 1-5.

5. Feng DD, Michaud JP, Li P, Zhou ZS, Xu ZF. 2015. The native ant, *Tapinoma melanocephalum*, improves the survival of an invasive mealybug, *phenacoccus solenopsis*, by defending it from parasitoids. Scientific Reports. 5: 15691.

6. Hanchinal SG, Patil BV, Basavanagoud K, Nagangoud A, Biradar DP, Janagoudar BS. 2011. Incidence of invasive mealybug (*Phenacocccus solenopsis* Tinsley) on cotton. Karnataka Journal of Agriculture Science. 24(2): 143-145.

7. Hodgson C, Abbas G, Arif MJ, Saeed S, Karar H. 2008. *Phenacoccus solenopsis* Tinsley (Sternorrhyncha: Coccoidea: Pseudococcidae), an invasive mealybug damaging cotton in Pakistan and India, with a discussion on seasonal morphological variation. Zootaxa. 1913: 1-35.

8. Ibrahim SS., Moharum FA., EL-GHany NMA. 2015. The Cotton mealybug *Phenacoccus solenopsis* Tinsly (Hemiptera: Pseudococcidae) as a new insect pest on tomato plants in Egypt. Journal of plant protection research. 55(1): 48-51.

9. Kaydan MB., Caliskan AF., Ulusoy MR. 2013. New record of invasive mealybug *Phenacoccus solenospis* Tinsley (Hemiptera: Pseudococcidae) in Turkey. EPPO Bulletin, 43(1): 169-171.

10. Khan HAA, Sayyed AH, Akram W, Raza S, Ali M. 2012. Predatory potential of *Chrysoperla carnea* and *Cryptolaemus montrouzieri* larvae on different stages of the mealybug, *Phenacoccus solenopsis*: A threat to cotton in South Asia. Journal of Insect Science 12:147.

11. Ma J, Hu XW, Peng ZQ, Liu HJ, Liang F, Lu YY. 2011. The potential geographical distribution of *Phenacoccus solenopsis* Tinsley based on the CLIMEX in China. Plant Quarantine. 25 (1): 5-8.

12. Miller DR. 2005. Selected scale insect groups (Hemiptera: Coccoidea) in the southern Region of the United States. Florida Entomologist, 88(4): 482-501.

13. Muniappan R., Shepard BM., Watson GW., Carner GR., Rauf A., Sartiami D., Hidayat P., Afun JVK., Goergen G., Ziaur Rahman AKM. 2009. New Records of invasive insects (Hemiptera: Sternorrhyncha) in Southeast Asia and West Africa. J. Agric. Urban Entomol. 26(4): 167-174.

14. Perez-Gelabert DE. 2008. Arthropods of Hispaniola (Dominican Republic and Haiti): A checklist and bibliography. Zootaxa 1831: 1-530.

15. Sahar M. Beshr, Suzan A. Badr, Anas A. Ahmad and G.H. Mohamed, 2016. New record of host plants of invasive mealybug *Phenacoccus solenopsis* Tinsley (Tinsley, 1898), (Hemiptera: Pseudococcidae) in Alexandria and Behaira governorates. J. Entomol., 13: 155-160.

16. Tanwar RK., Jeyakumar P., Singh A., Jafri AA., Bambawale OM. 2011. Survey for cotton mealybug, *Phenacoccus solenopsis* (Tinsley) and its natural enemies. Journal of Environmental biology. 32: 381-384.

17. Vennila S., Prasad YG., Prabhakar M., Agarwal M., Sreedevi G., Bambawale OM. Weed hosts of cotton mealybug, *Phenacoccus solenopsis* Tinsley (Hemiptera: Pseudococcidae). Journal of Environmental biology. 34: 153-158.

18. Wang YP., Watson GW., Zhang RZ. 2010. The potential distribution of an invasive mealybug *Phenacoccus solenopsis* and its threat to cotton in Asia. Agricultural and Forest Entomology, 1-14.

19. Wang YP, Wu SA, Zhang RZ. 2009. Pest risk analysis of a new invasive pest, *Phenacoccus solenopsis*, to China. Chinese Bulletin of Entomology. 46(1): 101-106.

20. Zhou A, Lu Y, Zeng L, Xu Y, Liang G (2012) Does Mutualism Drive the Invasion of Two Alien Species? The Case of *Solenopsis invicta* and *Phenacoccus solenopsis*. PLoS ONE 7(7): e41856.

21. Zhang P, Zhu X, Huang F, Liu Y, Zhang J, et al. (2011) Suppression of Jasmonic Acid-Dependent Defense in Cotton Plant by the Mealybug *Phenacoccus solenopsis*. PLoS ONE 6(7): e22378.
