# Supplementary material for: Niche shifts and the potential distribution of Phenacoccus solenopsis (Hemiptera: Pseudococcidae) under climate change
Source: PLoS One. 2017 Jul 10;12(7):e0180913. doi: 10.1371/journal.pone.0180913 (PMC5507313; doi:10.1371/journal.pone.0180913)
Supplement: S3 Table — (DOC) [file pone.0180913.s003.doc]

| 1 | 1 |  |  |  |  |  |  |  |  |  |  |  |  |  |  |  |  |  |  |
| --- | --- | --- | --- | --- | --- | --- | --- | --- | --- | --- | --- | --- | --- | --- | --- | --- | --- | --- | --- |
| 2 | .108^*^ | 1 |  |  |  |  |  |  |  |  |  |  |  |  |  |  |  |  |  |
| 3 | .499^**^ | .423^**^ | 1 |  |  |  |  |  |  |  |  |  |  |  |  |  |  |  |  |
| 4 | -.667^**^ | .046 | -.813^**^ | 1 |  |  |  |  |  |  |  |  |  |  |  |  |  |  |  |
| 5 | .598^**^ | .572^**^ | .081 | .046 | 1 |  |  |  |  |  |  |  |  |  |  |  |  |  |  |
| 6 | **.859^**^** | -.192^**^ | .606^**^ | **-.900^**^** | .188^**^ | 1 |  |  |  |  |  |  |  |  |  |  |  |  |  |
| 7 | -.486^**^ | .494^**^ | -.529^**^ | **.877^**^** | .368^**^ | -.844^**^ | 1 |  |  |  |  |  |  |  |  |  |  |  |  |
| 8 | .663^**^ | .066 | .133^*^ | -.217^**^ | .494^**^ | .438^**^ | -.145^**^ | 1 |  |  |  |  |  |  |  |  |  |  |  |
| 9 | .783^**^ | .271^**^ | .653^**^ | -.734^**^ | .448^**^ | .783^**^ | -.496^**^ | .250^**^ | 1 |  |  |  |  |  |  |  |  |  |  |
| 10 | .729^**^ | .221^**^ | -.046 | -.017 | **.900^**^** | .370^**^ | .142^**^ | .681^**^ | .439^**^ | 1 |  |  |  |  |  |  |  |  |  |
| 11 | **.920^**^** | .056 | .700^**^ | **-.893^**^** | .358^**^ | **.964^**^** | -.717^**^ | .506^**^ | .848^**^ | .464^**^ | 1 |  |  |  |  |  |  |  |  |
| 12 | .006 | -.774^**^ | -.291^**^ | -.154^**^ | -.426^**^ | .254^**^ | -.473^**^ | .026 | -.150^**^ | -.144^**^ | .073 | 1 |  |  |  |  |  |  |  |
| 13 | .284^**^ | -.510^**^ | -.008 | -.374^**^ | -.163^**^ | .442^**^ | -.508^**^ | .183^**^ | .126^*^ | .048 | .351^**^ | .816^**^ | 1 |  |  |  |  |  |  |
| 14 | -.364^**^ | -.614^**^ | -.533^**^ | .282^**^ | -.431^**^ | -.190^**^ | -.056 | -.178^**^ | -.449^**^ | -.248^**^ | -.360^**^ | .677^**^ | .241^**^ | 1 |  |  |  |  |  |
| 15 | .661^**^ | .468^**^ | .463^**^ | -.401^**^ | .607^**^ | .431^**^ | -.076 | .473^**^ | .586^**^ | .544^**^ | .596^**^ | -.353^**^ | .149^**^ | -.718^**^ | 1 |  |  |  |  |
| 16 | .249^**^ | -.572^**^ | -.067 | -.342^**^ | -.209^**^ | .423^**^ | -.515^**^ | .165^**^ | .078 | .024 | .312^**^ | **.882^**^** | **.977^**^** | .318^**^ | .060 | 1 |  |  |  |
| 17 | -.368^**^ | -.633^**^ | -.560^**^ | .305^**^ | -.423^**^ | -.199^**^ | -.043 | -.176^**^ | -.469^**^ | -.234^**^ | -.374^**^ | .691^**^ | .254^**^ | **.991^**^** | -.725^**^ | .333^**^ | 1 |  |  |
| 18 | -.088 | -.692^**^ | -.340^**^ | -.027 | -.499^**^ | .106 | -.373^**^ | .127^*^ | -.265^**^ | -.197^**^ | -.055 | .849^**^ | .616^**^ | .571^**^ | -.350^**^ | .712^**^ | .580^**^ | 1 |  |
| 19 | -.287^**^ | -.536^**^ | -.319^**^ | .126^*^ | -.400^**^ | -.066 | -.156^**^ | -.324^**^ | -.215^**^ | -.271^**^ | -.232^**^ | .547^**^ | .180^**^ | .746^**^ | -.624^**^ | .235^**^ | .753^**^ | .345^**^ | 1 |
|  | 1 | 2 | 3 | 4 | 5 | 6 | 7 | 8 | 9 | 10 | 11 | 12 | 13 | 14 | 15 | 16 | 17 | 18 | 19 |

Table S1 Correlation analysis of environmental variables

**: significant at the 0.05 level *: significant at the 0.1 level
